# Serum Anti-Zta and Anti-LMP1 Antibodies in Oropharyngeal Cancer Related to Epstein–Barr Virus—Diagnostic Usefulness

**DOI:** 10.3390/cancers16020341

**Published:** 2024-01-13

**Authors:** Anna Polz, Kamal Morshed, Bartłomiej Drop, Andrzej Drop, Małgorzata Polz-Dacewicz

**Affiliations:** 1 Genomed S.A., 02-971 Warsaw, Poland; anna.polz@genomed.pl; 2 Department of Otolaryngology Head and Neck Cancer, University of Technology and Humanities in Radom, 26-600 Radom, Poland; k.morshed@uthrad.pl; 3 Department of Computer Science and Medical Statistics with the e-Health Laboratory, 20-090 Lublin, Poland; bartlomiej.drop@umlub.pl; 4 1st Department of Medical Radiology, Medical University of Lublin, 20-093 Lublin, Poland; andrzej.drop@umlub.pl; 5 Department of Virology with Viral Diagnostics Laboratory, Medical University of Lublin, 20-093 Lublin, Poland

**Keywords:** anti-Zta antibody, anti-LMP1 antibody, oropharyngeal cancer, EBV

## Abstract

**Simple Summary:**

Unlike NPC, there is little research concerned with the role of EBV in the development of OPSCC. Therefore, it is necessary to search for new markers enabling the early diagnosis and prognosis of this disease. The aim of this study was to evaluate the diagnostic utility of anti-Zta and anti-LMP1 antibodies in EBV positive OPSCC patients. For this purpose, experiments were carried out to determine both the prevalence and level of anti-Zta and anti-LMP1 antibodies in serum patients with OPSCC in relation to grading and TNM classification. The highest level of tested antibodies was observed in more advanced clinical stages. The diagnostic accuracy of anti-Zta antibodies was confirmed using ROC analysis. We showed that the determination of both types of antibodies may be useful in the diagnosis of OPSCC. Moreover, anti-LMP1 antibody may be a diagnostic as well as prognostic biomarker.

**Abstract:**

Background: The role of the Epstein–Barr virus (EBV), the first known human oncogenic virus, in the development of nasopharyngeal cancer (NPC) is already well documented. There are few studies in the available scientific literature on oropharyngeal cancer associated with EBV infection. Due to the lack of an effective vaccine against EBV, it is necessary to search for new markers for the early diagnosis and prognosis of this disease. The aim of current study was to determine the usefulness of anti-Zta and anti-LMP1 antibodies as diagnostic and prognostic markers in EBV positive OPSCC patients. Methods: For this purpose, experiments were carried out to determine both the prevalence and level of EBVCA, EBNA1, EA, Zta, and LMP1 antibodies in serum patients depending on histological differentiation-grading and TNM classification (ELISA assay). Results: Based on the obtained results, we showed that OPSCC EBV positive patients are characterized by a higher level of anti-Zta antibodies than in the EBV negative group. Their level depended on the clinical stage. Moreover, a ROC analysis confirmed the diagnostic accuracy of anti-Zta antibodies. Conclusions: Anti-Zta and anti-LMP1 antibodies may be useful in the diagnosis of OPSCC. It seems that combined antibody testing should be performed to increase diagnostic accuracy.

## 1. Introduction

Head and neck cancers (HNCs), an important clinical and epidemiological problem, show an increasing trend in both incidence and mortality. In Poland, about 5000 new HNC cases are registered every year [1]. Most HNCs arising from the epithelium of the oral cavity, pharynx, and larynx are classified as head and neck squamous cell carcinoma (HNSCC). In the multifactorial etiology of these cancers, persistent infections with oncogenic viruses, especially human papillomavirus (HPV) and EBV, also play an important role [2,3].

Epstein–Barr virus (EBV), also known as human herpes virus 4 or human lymphocryptovirus gamma 4, was recently classified in the genus *Lymphocryptovirus* as a member of the *Orthoherpesviridae* family, subfamily *Gammaherpesvirinae* [4].

Primary infection usually occurs in childhood and is quite often asymptomatic. The virus is transmitted through saliva and other body fluids as well as during organ transplantation from an EBV positive recipient [5].

Epstein–Barr virus (EBV) is widespread, with 95% of the world’s human population infected. It is also the first known human virus with carcinogenic potential [6]. EBV infection may lead to the development and progression of various B-cell cancers, e.g., Burkitt’s lymphoma and Hodgkin’s lymphoma, but may also derive from epithelial cells, e.g. in gastric cancer and nasopharyngeal cancer (NPC) [7,8,9,10,11,12,13]. Additionally, cases of breast cancer, thyroid cancer, salivary gland cancer, and liver and bile duct cancer have been described [5].

Studies available in the medical literature only concern NPC. However, the NPC shows geographical and ethnic diversity. Moreover, most available studies concern the Asian population, while there are few such studies in other populations. This tumor is particularly common in South China, where the risk of developing this cancer may be up to 50 times higher compared to non-endemic areas. In other parts of the world, this cancer is rare [14].

There are few studies in the available scientific literature on oropharyngeal cancer associated with EBV infection [3]. However, among head and neck cancers in Poland, oropharyngeal cancer dominates. The oropharynx and the nasopharynx are different anatomical regions. According to the Globocan registry, in 2020, 1659 new cases of oropharyngeal cancer, followed by hypopharyngeal cancer (1126), and only 297 cases of NPC, were registered in Poland [15].

Like other herpesviruses, EBV establishes a latent infection, reactivated periodically into the lytic cycle, which plays an important role in the pathogenesis of EBV related malignancies [16]. The scientific literature provides increasing evidence that EBV lytic-phase proteins play a pivotal role in the oncogenesis [17,18]. In this phase, many proteins are expressed, which are classified as: immediate early (IE), early (E), and late (L) lytic proteins. Epstein–Barr nuclear antigen 1 (EBNA1), Epstein–Barr virus-encoded RNAs 1 and 2 (EBER1 and EBER2), and BamHI-A rightward transcripts (BART) as well as latent membrane proteins 1 and 2 (LMP1, LMP2) belong to this group of specific proteins.

EBNA1, a DNA binding responsible for viral replication, is the only protein expressed in all EBV-positive tumors, and, sometimes, the only protein expressed at all [19]. It maintains the EBV genome in latently infected cells but may also contribute to cell immortalization and neoplastic transformation by interfering with tumor suppressors, inducing DNA damage and changing signaling pathways [10]. Moreover, EBNA 1 is a transcription activator and increases the expression of LMP1.

LMP1, a transmembrane protein, is the product of the BLNF1 gene [7]. LMP1 influences many different cellular mechanisms, which ultimately lead to promoting oncogenic transformation, proliferation, angiogenesis, metastasis, and invasion, as well as the development of the tumor microenvironment [19]. Due to its important role in cancer development and progression, LMP1 is considered a prognostic marker in NPC [20,21].

In the early lytic phase, the genes of proteins involved in viral DNA replication are expressed primarily. The first protein in the virus’s lytic cycle is the Z protein, a product of the BZLF1 gene, also called Zta or ZEBRA [22,23]. In the late phase of infection, proteins involved in the construction of the capsid and the formation of new virions are synthesized.

The presence and serum levels of specific antibodies against capsid antigen (EBVCA) in the IgM and IgG classes, nuclear antigen (EBNA), and early antigen (EA) are used in the routine diagnosis of EBV infections. As shown in many studies, higher levels of anti-EBVCA, anti-EA, and anti-EBNA antibodies are detected in the serum of NPC patients [24,25,26,27,28]. The serum levels of anti-EBVCA antibodies in the Ig A class were established as one of the indicators for screening patients for NPC [29]. Moreover, the expression of latent genes in EBV-infected states was also discovered [30].

In recent years, many researchers have made efforts to identify diagnostic and prognostic factors in various cancers, while searching for the relationship between these biomarkers and the clinical stage of cancer. Therefore, in the current study, we aimed to examine the serum prevalence and the level of anti-Zta and anti-LMP1 antibodies, both in IgA and IgG classes, in EBV-positive and EBV-negative OPSCC patients, as well as in non-cancer individuals. We tried to check whether these antibodies could serve as diagnostic and/or prognostic biomarkers in oropharyngeal cancer. For this purpose, the relationship between antibody levels, TN classification, and histological differentiation (grading) was assessed.

## 2. Materials and Methods

### 2.1. Patients

The study involved 110 patients with diagnosed and histopathologically confirmed squamous cell carcinoma of the oropharynx (OPSCC), hospitalized at the Department of Otolaryngology, Head and Neck Cancer, University of Technology and Humanities in Radom, Poland. Patients were assigned to a study group based on a negative result of the p16 immunohistochemical screening test, which was further verified using PCR. All patients had not received radiotherapy or chemotherapy before.

The control group consisted of 40 patients of the outpatient clinic in whom cancer was excluded. The control group patients matched in terms of sociodemographic features.

### 2.2. Clinical Specimens

Tissue and blood were collected from all cancer patients. Only blood was collected from the control group. HPV DNA and EBV DNA were detected in tumor tissue. However, only anti-EBV antibodies were detected in the serum.

#### 2.2.1. Tissue Samples Collection

Tissue samples collected from all patients during the surgery were frozen at −80 °C and stored until analysis. During primary diagnosis, the classification of the tumor, node, and metastases (TNM) was determined according to the eighth edition of the TNM classification of head and neck cancer [31,32,33]. Histological grading was performed according to the World Health Organization criteria, which divide tumors into three types: well differentiated (G1), moderately differentiated (G2), and poorly differentiated (G3) [34].

#### 2.2.2. Serum Collection

Venous blood samples collected from all patients were centrifuged at 1500 rpm for 15 min at room temperature, and the sera were frozen at −80 °C until analysis.

### 2.3. Molecular Methods

#### 2.3.1. DNA Extraction and Detection

A fragment (20 mg) of freshly frozen oropharyngeal squamous cell carcinoma (OPSCC) tissue was cut and then homogenized using a manual homogenizer (Omni TH/Omni International/Kennesewa, GA, USA). DNA was extracted using the QIAampDNA Mini Kit (Qiagen, Hilden, Germany) according to the manufacturer’s recommendation. The DNA isolated in this way was stored at −20 °C until the test was performed. In order to check the quality of the obtained DNA (presence of Polymerase Chain Reaction Inhibitors), β-globin was determined.

#### 2.3.2. HPV Detection

To detect and determine the HPV genotype, the commercially available INNO-LiPA HPV Genotyping Extraassay/Innogenetics, Gent, Belgium diagnostic kit was used. This kit is based on the amplification of a 65-bp fragment from the L1 region of the HPV genome using the SPF10 primer set. The PCR products are then typed using a reverse hybridization method.

#### 2.3.3. EBV Detection

EBV DNA was detected using the commercially available Gene Proof EBV diagnostic kit (Brno, Czech Republic) according to the manufacturer’s instructions. All samples and negative controls (DNA elution buffer) were analyzed in duplicate. A specific conserved DNA sequence for the EBV nuclear antigen 1 gene (EBNA-1) was amplified using Light Cycler 2.0 Software Version 4.1. (Roche Applied Science System, Penzberg, Germany).

### 2.4. Serological Methods

To detect antibody levels, serological tests were conducted using the ELISA method. Designed antibodies: anti-VCA IgM, anti-VCA IgG, and anti-EBNA IgG (NovaLisa Epstein–Barr Virus EBNA IgG/Nova Tec Imunodiagnostica GmbH/Hessen, Germany). The NovaTec Epstein–Barr virus (EBV) IgG-ELISA was used for the qualitative evaluation of IgG class antibodies against the Epstein–Barr virus. Samples were considered positive if the absorbance value was higher than 10% over the cut-off. This test was used to assess the prevalence of EBV antibodies.

Anti-EA IgG antibodies were detected using (ELISA-VIDITEST anti-EA (D) EBV IgG/Vidia, Czech Republic). All tests were performed according to the manufacturer’s recommendations. ELISA-VIDITEST anti-EA is semiquantitative test. Samples with absorbances higher than 110% of the cut-off value are considered positive.

Serum anti-LMP1 and anti-Zta antibodies levels were determined using the commercially available Microblot-Array test (TestLine Clinical Diagnostics Ltd., Brno, Czech Republic) according to the manufacturer’s instructions. The MICROBLOT-ARRAY kits (CE IVD) were optimized and validated for the detection of IgA, IgG, and IgM antibodies in human serum, plasma, or cerebrospinal fluid. This test contained a combination of selected parts of the specific antigens of EBV (EBNA-1, EBNA-2, VCA p18, VCA p23, EA-D p54, EA-D p138, EA-R, Rta, ZEBRA, gp85, gp350, and LMP1).

### 2.5. Statistical Methods

Tibco Statistica 13.3 (StatSoft, Kraków, Poland) and GraphPad Prism software version 10.1.1. (San Diego, CA, USA) were used to perform the data analyses. The Shapiro–Wilk test was used to test for a normal distribution of continuous variables. The relationship between clinical and demographic parameters was calculated using Pearson’s chi-square test. All analyzed parameters were presented as arithmetic means and standard deviations (SD) as well as median, lowest, and highest values. To compare differences between studied groups, the Mann–Whitney U test and/or Kruskal–Wallis Test were used. The correlation between levels of anti-Zta and anti-EA antibodies, both in the IgA and IgG classes, was assessed using the Spearman correlation rank test. To determine the diagnostic accuracy of serum anti-Zta IgA antibody levels in OPSCC patients who were EBV positive, the receiver operating characteristic curve (ROC) analysis was performed.

## 3. Results

The research group of patients (*N* = 110) consisted of 58 subjects with EBV DNA detected in the tumor tissue, hereinafter referred to as EBV positive—EBV(+), and 52 individuals without EBV DNA detected, hereinafter referred to as EBV negative—EBV(−). Two age groups were distinguished, i.e., 50–59 and 60–79. The average age of the patients in the study group was 54.7 (SD = 2.6) and 68.5 (SD = 5.5), respectively. The sociodemographic and clinical characteristics of the study group and control group are presented in Table 1.

None of the EBV positive or EBV negative patients had distant metastases. Both groups did not differ significantly in terms of sociodemographic and clinical characteristics. Therefore, they had no effect on the analyzed parameters.

### 3.1. Evaluation of the Serum Prevalence of Selected Anti-EBV in Oropharyngeal Cancer Patients in Comparison to the Control Group

In the first stage of our research, a screening test was carried out to determine what type of antibodies were present in the serum of the examined subjects. The analysis included those antibodies that were detected in the highest percentage of subjects. Therefore, the following antibodies were analyzed: anti-EBNA1, EBVCA p18 (marked in the article as EBVCA), EA-D p138 (named in the article as EA), as well as anti-Zta and anti-LMP1, both in IgA and IgG classes.

As shown by the data in Table 2, both in the group of patients with oropharyngeal cancer and in the control group, the presence of EBVCA IgG antibodies and EBNA IgG antibodies was detected. Both anti-EBVCA (in class IgA) and anti-EBNA (in class IgA) antibodies were detected only in EBV positive patients with oropharyngeal cancer. Anti-EA antibodies were also found in the serum of OPSCC patients in both the IgA and IgG classes. Anti-VCA antibodies in the IgM class were not found in any studied groups.

#### Evaluation of the Serum Level of Selected Anti-EBV Antibodies in Oropharyngeal Cancer Patients in Comparison to the Control Group

From the results obtained in our research, it can be concluded that statistically significant differences in the level of antibodies between the studied groups concerned EBVCA and EBNA antibodies in the IgG class. These results are presented in Table 3. They achieved the highest titer in the group of EBV positive OPSCC patients and were 775.2 U/mL and 487.0 U/mL (*p* < 0.0001), respectively. Moreover, among the OPSCC EBV positive patients, a quite high titer of EBNA1 antibodies in the IgG class was observed—487.0 U/mL, but much higher was observed in EBNA in the IgA class—683.2 U/mL.

### 3.2. The Frequencies of Anti-EBNA IgA, EBNA IgG, EBVCA IgA, and EBVCA IgG in OPSCC Patients in Relation to Grade (G1–G3)

Then, only EBV positive OPSCC patients were further analyzed. In the next stage of our analysis, the incidence of antibodies was assessed depending on grading and TNM classification. The results obtained are presented in Table 4. As the analysis has shown, the frequency of anti-EBNA in the IgA and IgG classes’ antibodies depended on the grading. They were most often detected in stages G2 and G3.

#### The Frequencies of Anti-EBNA IgA, EBNA IgG, EBVCA IgA, and EBVCA IgG Antibodies in EBV Positive OPSCC Patients Depending on the TNM Classification

This assessment is presented in Table 5. The prevalence of EBNA IgA and IgG depended on the T and N characteristics. In the case of EBVCA IgA, no such relationship was found. In turn, EBVCA IgG antibodies were the most detected at advanced stages (T4 and N3).

### 3.3. Detailed Assessment of the Significance of Anti-Zta and Anti-LMP1 Antibodies in Oropharyngeal Cancer Linked to EBV Infection

In this part of the analysis, the frequency of antibody detection was compared in the group of EBV-positive and EBV-negative OPSCC patients. The obtained results are presented in Table 6. Anti-Zta antibodies were detected significantly more often in the group of patients infected with EBV, both in the IgA and IgG classes. However, anti-LMP1 IgA and IgG antibodies were detected only in the OPSCC group of EBV positive patients.

#### 3.3.1. Evaluation of the Frequencies and Serum Levels of Anti-Zta and Anti-LMP1 in Oropharyngeal Cancer Patients Depending on the Grading (G1–G3) and TNM Classification

##### The Frequencies of Anti-Zta and Anti-LMP1 in Oropharyngeal Cancer Patients Depending on the Grading (G1–G3)

The frequency of anti-Zta and anti-LMP1 antibodies was analyzed, both in the IgA and IgG classes, as shown in Table 7. The prevalence of anti-Zta IgA and IgG antibodies depended on the grading in a statistically significant way. They were detected more often in the third grade. In turn, only the expression of anti-LMP1 antibodies in the IgG class depended on grading.

##### The Frequencies of Anti-Zta and Anti-LMP1 in Oropharyngeal Cancer Patients Depending on the TNM Classification

The next point of our analysis was to assess the frequency of anti-Zta and anti LMP1 antibodies in relation to the TNM classification (Table 8). As indicated by the obtained results, anti-Zta IgA antibodies were most often detected at the advanced stages (T4 and N3). However, only anti-LMP1 IgG antibodies were more frequently detected in stage N3.

##### The Serum Level of Anti-Zta and Anti-LMP1 in Oropharyngeal Cancer Patients Depending on the Grading (G1–G3) and TNM Classification

**Figure 1:**
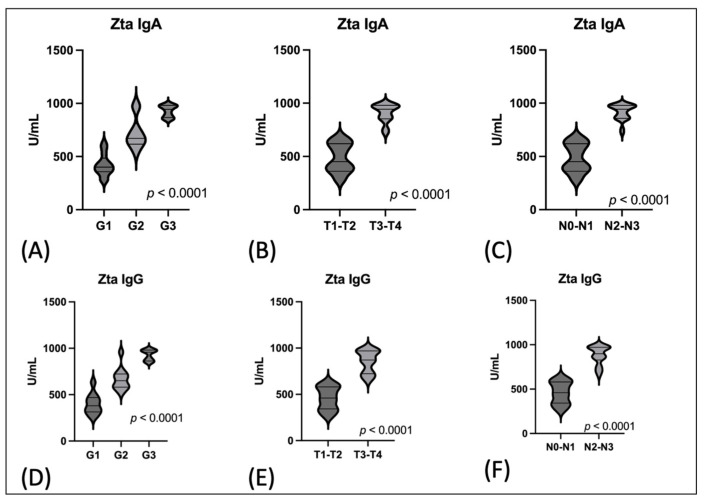
The serum level of Zta antibodies in EBV positive OPSCC: (**A**) the serum level of anti-Zta IgA antibodies in relation to grading; (**B**) the serum level of anti-Zta IgA antibodies in relation to T stages; (**C**) the serum level of anti-Zta IgA antibodies in relation to N stages; (**D**) the serum level of anti-Zta IgG antibodies in relation to grading; (**E**) the serum level of anti-Zta IgG antibodies in relation to T stages; (**F**) the serum level of anti-Zta IgG antibodies in relation to N stages.

In the next stage, the level of anti-Zta antibodies was assessed depending on the degree of tumor differentiation and TN classification. Due to the small number of cases in individual groups T and N, in order to assess the level of antibodies, the groups were combined and analyzed as T1-T2 and T3-T4, and N0-N1 and N2-N3. This relationship was highly statistically significant (*p* < 0.0001). These results are illustrated in Figure 1. The highest levels of antibodies were observed at advanced stages of the disease. This concerned both the IgA and IgG classes of anti-Zta antibodies. 

**Figure 2:**
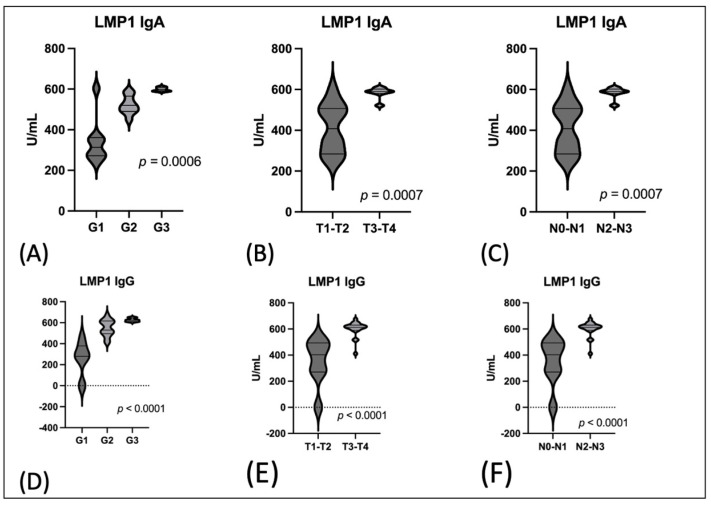
The serum level of LMP1 antibodies in EBV positive OPSCC: (**A**) the serum level of anti-LMP1 IgA antibodies in relation to grading; (**B**) the serum level of anti-LMP1 IgA antibodies in relation to T stages; (**C**) the serum level of anti-LMP1 IgA antibodies in relation to N stages; (**D**) the serum level of anti-Zta IgG antibodies in relation to grading; (**E**) the serum level of anti-LMP1 IgG antibodies in relation to T stages; (**F**) the serum level of anti-LMP1 IgG antibodies in relation to N stages.

A similar relationship was demonstrated regarding the level of anti-LMP1 antibodies. Both levels in the IgA and IgG classes were higher in G3 and T3-T4 as well as in cases of N2-N3 (Figure 2). 

### 3.4. Correlation between the Level of All Tested Antibodies with Particular Emphasis on Anti-Zta and Anti-EBNA1 Antibodies in Serum of EBV Positive OPSCC Patients

**Figure 3:**
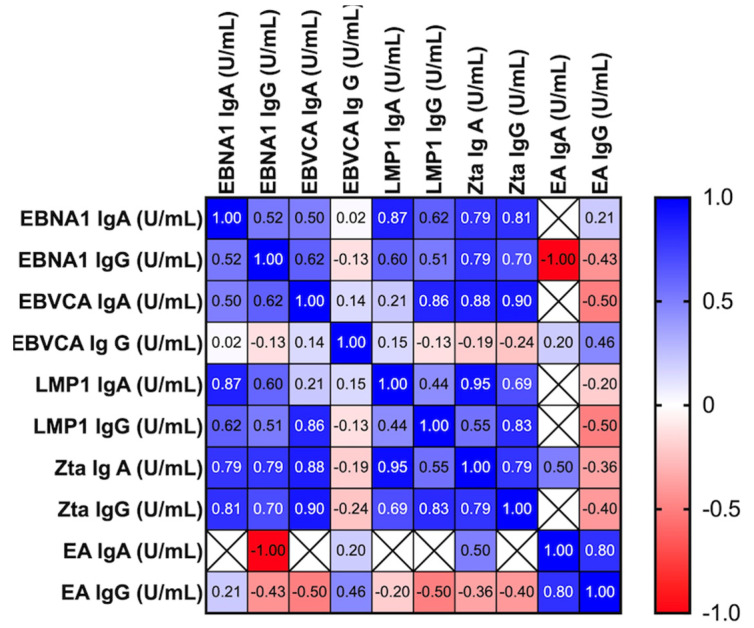
Correlation between the level of tested antibodies in EBV positive OPSCC patients. Spearman’s rank coefficients are presented as the intensity of the colors. The closer Rs is to +1 or −1, the stronger the correlation. A perfect positive correlation is +1 (blue color), and a perfect negative correlation is −1 (red color).

Considering the possible correlation between the level of anti-Zta IgA and anti-EBNA1 IgA antibodies, after statistical analysis using multiple linear regression, their high correlation was demonstrated (Figure 3). However, no correlation was found between the levels of anti-Zta and anti-EA antibodies, both in the IgA and IgG classes.

In the EBV positive OPSCC patients, a strong positive correlation was detected between:

1. The concentration of EBNA1 IgA and Zta IgA *p* < 0.0001; Zta IgG *p* < 0,0001; LMP1 IgG *p* < 0.0001; LMP1 IgA *p* = 0.0001; EBNA1 IgG *p* = 0.0001;
2. The level of anti-Zta antibodies both in the IgA and IgG classes and anti-LMP1 IgA and IgG *p* < 0.0001. We also observed a correlation between the level of EBNA1 IgA and EBNA1 IgG *p* = 0.03; LMP1 IgA *p* = 0.007; LMP1IgG *p* = 0.02. However, they are less important for the issue at hand. The level of EA antibodies did not show any relationship with any of the tested parameters.

### 3.5. Receiver Operating Characteristic (ROC) Curve Analysis to Determine the Diagnostic Accuracy of Serum Anti-Zta IgA Antibody Level in OPSCC Patients EBV Positive vs. OPSCC Patients EBV Negative

**Figure 4:**
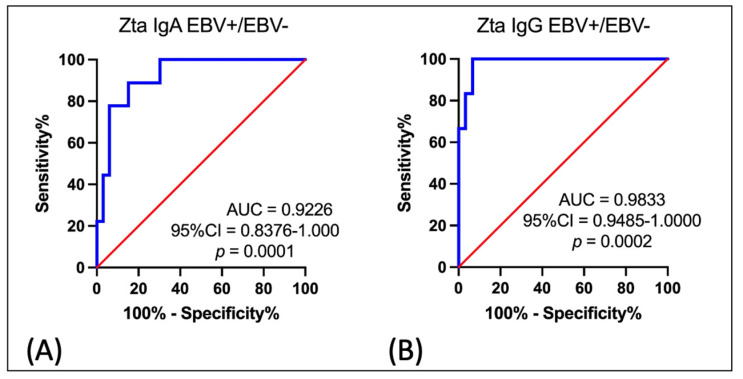
The receiver operating characteristic (ROC) analysis for anti-Zta IgA (**A**) and anti-Zta IgG (**B**) antibodies. As shown by the area under the curve (AUC), the level of both antibody types was the most sensitive and specific parameter to determine EBV positive OPSCC patients. Blue line—serum anti-Zta antibodies (U/mL).

In the last stage of our research, we tried to check the accuracy of anti-Zta antibodies, i.e., whether the serum anti-Zta antibodies can serve as a good diagnostic biomarker in the diagnosis of patients with OPSCC. For this purpose, an ROC curve analysis was used to compare the levels of anti-Zta antibodies in the IgA class as well as in the IgG class in the serum of EBV positive OPSCC patients with the group of EBV negative patients (Figure 4). As the area under the curve (AUC) shows, the level of anti-Zta IgA antibodies (Figure 4A) was the sensitive and specific parameter to determine patients with EBV positive OPSCC (AUC = 0.9226; Std. Error 0.0434; 95% CI 0.8376–1.000; *p* = 0.0001). Similar results were obtained for anti-Zta antibodies in the IgG class (Figure 4B) (AUC 0.9833; Std. Error 0.01779; 95% CI 0.9485–1.000; *p* = 0.0002).

The obtained results confirm the diagnostic accuracy for anti-Zta antibodies. The analysis shows that the determination of both types of antibodies may be useful in the diagnosis of OPSCC linked to EBV.

## 4. Discussion

The association between EBV and the development of nasopharyngeal cancer is well documented [10,11,12,13,14]. In our team’s previous research on the prevalence of oncogenic viruses in oropharyngeal cancer (OPSCC), EBV was detected in 53.3% of cases and HPV in 26.7% of cases [35].

Our research and that of other authors have demonstrated the presence of EBV in oropharyngeal cancer, although these studies are few [3,35,36]. As mentioned in the introduction, oropharyngeal cancer is not a common cancer in our country. To the best of our knowledge, these are the first studies of this kind in the Polish population; hence, our decision to undertake such research. There are no studies evaluating the level of these antibodies in oropharyngeal cancer associated with EBV infection.

On the other hand, the basis of modern oncology is personalized targeted therapy. Therefore, new cancer biomarkers with a positive predictive value are constantly being sought. In recent years, many researchers have made efforts to identify diagnostic and prognostic factors in various cancers, while searching for the relationship between these biomarkers and the clinical stage of cancer.

As numerous studies have shown, patients with NPC have an increased level of antibodies against several antigens of the EB virus, i.e., viral capsid antigen (VCA), early antigen (EA), and EB nuclear antigen (EBNA), which have been used in clinical diagnostics [26,27]. Many studies have indicated that the detection of anti-EBV antibodies in patients’ serum is not only a useful marker in early diagnosis, but also in monitoring the recurrence and progression of cancers associated with EBV infection. As many researchers emphasize, this antibody assessment is particularly important in patients with NPC, especially in high-risk areas [37]. Moreover, they point out that the assessment of serum EBV antibodies has a high diagnostic accuracy in early stage NPC. According to Liu et al. [38], these observations do not depend on the size of the research group and ethnicity. Due to the small number of studies in non-Asian populations, our research is absolutely justified.

### Criteria Qualifying Patients for the Research Group

In the studies presented, we assessed the diagnostic utility of anti-Zta and anti-LMP1 antibodies in EBV positive OPSCC patients. For this purpose, experiments were carried out to determine both the prevalence and level of these antibodies in serum patients with OPSCC. However, patients for the study were selected based on the presence of EBV DNA in the tumor tissue—this was the basic qualifying criterion for the study.

In turn, the exclusion criterion was the presence of HPV DNA in the tumor tissue. Only HPV negative patients were included in the study group. We followed the recommendations of the US Joint Committee on Cancer TNM 8th edition [31]. The US Joint Committee on Cancer TNM 8th edition recommends the stratification of all OPSCC cases by HPV status. Therefore, all samples collected from patients with oropharyngeal carcinoma were tested for HPV. The idea was to ensure that the presence of HPV was not a factor disturbing the obtained results.

As previously mentioned, EBV establishes a latency phase of infection during which only certain different proteins are synthesized. All types of latency express the nuclear antigen EBNA1.

Therefore, in the first stage of our study, we assessed both the frequency and level of anti-EBNA 1 antibodies in the IgG and IgA classes. Additionally, we evaluated the prevalence and level of anti-EBVCA antibodies. Both types of antibodies were more common in EBV-positive OPSCC subjects compared to EBV-negative patients. Both anti-EBVCA in class IgA (32.7%) and anti-EBNA in class IgA (36.2%) antibodies were detected only in EBV positive patients with oropharyngeal cancer. Our results demonstrated the highest titer of these antibodies in the group of EBV positive OPSCC patients. These differences were statistically significant. It is known that high titers of both types of antibodies indicate previous EBV infection. In turn, anti-EA antibodies were also found in the serum of OPSCC patients for both the IgA (8.6%) and IgG (29.3%) classes.

Only in the first stage of our study did we compare the prevalence of antibodies in the EBV+ and EBV− groups. In the further part of the analysis, we focused only on the group of patients with EBV-positive oropharyngeal cancer.

Many authors have demonstrated a relationship between serum viral capsid antigen (VCA-IgA) and early IgA (EA-IgA) titers and TNM classification in NPC cases. They emphasize that high titers of these antibodies in patients’ serum are significantly associated with an advanced clinical stage [25,26,27,28].

Elucidating the role of latent EBV genes synthesized in oropharyngeal cancers is important for understanding the role of viral infection in cancer development and progression in this as yet insufficiently studied location. For this purpose, the level of antibodies was analyzed depending on the grading and TNM classification.

Our research team’s results presented here indicate that the level of anti-EBNA 1 is much higher in the EBV positive OPSCC patients compared to the EBV negative and control groups. We observed higher titers of EBNA 1 in the IgA class in OPSCC patients at a more advanced clinical stage (T3-T4 and N2-N3). The obtained results indicated that the frequency of anti-EBNA 1 in the IgA and IgG classes’ antibodies depended on the grading. They were most often detected in stages G2 and G3. All these differences we observed turned out to be statistically significant.

In the next stage of our own study, we analyzed anti-Zta antibodies. The first protein synthesized in the early lytic phase of the EBV life cycle is the Zta protein as a product of the BZLF1 gene. Many researchers have observed a high prevalence of anti-Zta IgG antibodies in patients with NPC, including those negative for anti-EBVCA as well as anti-EA, both for the IgG and IgA classes [39,40]. Zhang et al. [41] conducted an interesting systematic evaluation of the diagnostic value of serum anti-Zta antibody in NPC patients. The authors emphasized that the detection of VCA-IgA, EBNA1-IgA, and Rta-IgG has a high accuracy in the early diagnosis of NPC. On the other hand, they believe that EA IgA detection can be used for diagnosis but not for the screening of NPC.

Our research team’s results presented here indicate that anti-LMP1 and anti-Zta antibodies were detected only in EBV-positive patients. Their level depended on the clinical stage. The highest level of these antibodies was observed at advanced clinical stages, i.e., T3-T4 and N2-N3 (*p* < 0.0001). We found a similar relationship in regard to grading (G), i.e., in moderately differentiated (G2) and poorly differentiated (G3) tumors, the level of both anti-Zta and anti-LMP1 antibodies was the highest (*p* < 0.0001). Anti-EA antibodies, which may indicate the reactivation of the infection, were detected in a small percentage of the examined individuals. In contrast, anti-Zta antibodies in the IgA class were detected in 56.8% of EBV positive OPSCC cases and in the IgG class in 53.4% of cases.

He at al. [14] demonstrated higher frequencies of anti-EBNA1, LMP1, and Zta in the IgA class antibodies in the healthy population of endemic NPC regions compared to non-endemic regions. Moreover, many other researchers have found that the reactivation of the EBV infection and the associated higher levels of antibodies, especially in the IgA class, are present in patients several years before the diagnosis of NPC [29,42,43,44,45].

The EBNA1 protein is expressed at all stages of the viral replication cycle. Therefore, the determination of only anti-EBNA1 antibodies may have limited prognostic significance. Zta is the first protein of the lytic cycle. Therefore, the presence of anti-Zta antibodies in serum may indicate the lytic phase of EBV infection. Due to the fact that the antibody titer depended on the grading and was higher in more advanced stages of the disease, it can be considered as a diagnostic marker in OPSCC.

In our study, a correlation was found between the levels of anti-Zta and anti-EBNA1 antibodies, but no correlation was found between the levels of anti-Zta and anti-EA antibodies. For this reason, it seems that anti-Zta antibodies, especially in the IgA class, are a better indicator of the reactivation of the latent phase of EBV infection in patients with oropharyngeal cancer.

In our study, anti-LMP1 antibodies, against the main oncoprotein of EBV, were detected only in EBV positive OPSCC patients. The highest level of anti-LMP1 was observed in more advanced clinical stages. Therefore, it seems that anti-LMP1 antibody may be a diagnostic as well as prognostic biomarker.

Despite many attempts to develop a vaccine against EBV, none of the tested preparations has been approved for general use [46]. Therefore, without an effective preventive vaccine, it is necessary to search for markers that could be useful both in the early diagnosis and prognosis of diseases associated with EBV infection. For this reason, in the last step of our own research, we tried to check whether the serum anti-Zta antibodies can serve as a good diagnostic biomarker. For this purpose, the receiver operating characteristic (ROC) curve analysis was designed to determine the diagnostic accuracy of anti-Zta antibodies in EBV positive oropharyngeal cancer patients compared to EBV negative OPSCC. As the area under the curve (AUC) showed, the level of anti-Zta IgA (*p* = 0.0001) and anti-Zta IgG (*p* = 0.0002) antibodies was the sensitive and specific parameter to determine patients with EBV positive OPSCC.

A limitation of our study was that the group of patients too small. Therefore, due to the small number of cases in individual groups T (tumor size) and N (lymph nodes involvement), in order to assess the level of antibodies, the groups were combined and analyzed as T1-T2 and T3-T4, and N0-N1 and N2-N3. Therefore, further research is needed to verify the observed trend. Nevertheless, the obtained results may suggest that the level of antibodies increases with the progression of histological grade. Our results demonstrate the association of the frequency and level of tested antibodies in clinical stages of the EBV positive oropharyngeal cancer.

Our research and that of other authors have demonstrated coinfection HPV/EBV in HNCs [47,48]. According to Blanco et al. [48], HR-HPV/EBV coinfection could play a significant role as cofactors in the development of these malignancies. Both epidemiological and clinical evidence suggest that HR-HPV/EBV co-infection in the development of HNC requires further investigation. In the future, it would be worth assessing how EBV/HPV co-infection affects the analyzed parameters, which may be the subject of further research.

Summing up, the existing scientific studies demonstrate the usefulness of anti-Zta antibodies in the diagnosis of NPC. However, our results also indicate the usefulness of these antibodies as a non-invasive biomarker in the diagnosis of oropharyngeal cancer, confirmed through an ROC analysis. Nevertheless, these observations would require confirmation in a larger group of patients.

## 5. Conclusions

Head and neck cancers are an important problem from both a clinical and epidemiological point of view worldwide. A systematic increase in morbidity and mortality has been observed, and this trend will continue. This group includes tumors of various locations, including oropharynx. Due to the fact that it is not a common cancer in Europe, there are not many studies on this issue. We hope that our research will contribute to further research on the role of the EB virus and other oncogenic viruses in the development and possible progression of oropharyngeal cancer, especially when it comes to searching for biomarkers useful in diagnosis and prognosis.

The presented results indicate that the level of anti-EBNA1 was much higher in the EBV positive group. We showed a higher percentage of anti-Zta antibodies in patients with OPSCC. Furthermore, the accompanying significantly higher concentrations of these antibodies may suggest the reactivation of the EBV infection. Anti-LMP1 antibodies were detected only in EBV-positive patients. Their level depended on the clinical stage. The determination of specific IgA, IgG classes’ antibodies against particular EBV antigens may be a useful tool for the detection and determination of the stage of EBV infection. It seems that combined antibody testing should be performed to increase diagnostic accuracy. The obtained results confirm the usefulness of Zta IgA testing in OPSCC cases.

## Figures and Tables

**Table 1:** Baseline characteristics of research group (patients with oropharyngeal cancer) and control group.

		EBV				*p*	Total Patients		Control Group		*p*
		Positive		Negative							
		*N*	%	*N*	%		*N* = 110	%	*N* = 40	%	
Sex	Female	8	13.8	7	13.5	0.9999	15	13.8	6	15.0	0.7957
	Male	50	86.2	45	86.5		95	86.2	34	85.0	
Age	50–59	27	46.6	24	46.2	0.1116	59	53.4	21	52.5	0.9999
	60–79	31	53.4	28	53.8		51	46.6	19	47.5	
Place of residence	Urban	41	70.7	36	69.2	0.1667	77	70.7	28	70.0	0.9999
	Rural	17	29.3	16	30.8		33	29.3	12	30.0	
Smoking	Yes	38	65.8	35	67.3	0.8427	73	65.8	26	65.0	0.9999
	No	20	34.5	17	32.7		37	34.5	14	35.0	
Alcohol abuse	Yes	28	48.3	25	48.1	0.9834	53	48.3	19	47.5	0.9999
	No	30	51.7	27	51.9		57	51.7	21	52.5	
G	G1	19	32.8	17	32.7	0.9997					
	G2	30	51.7	27	51.9						
	G3	9	15.5	8	15.4						
T	T1	7	12.1	8	15.4	0.9505					
	T2	27	46.6	22	42.3						
	T3	16	27.6	15	28.8						
	T4	8	13.7	7	12.1						
	N0	23	39.7	22	42.3						
N	N1	11	19.0	10	19.2	0.9844					
	N2	14	24.1	11	21.2						
	N3	10	17.2	9	17.3						
M	M0	58	100.0	52	100.0						

**Table 2:** Prevalence of selected anti-EBV antibodies in EBV positive and EBV negative OPSCC patients compared to the control group (%).

Parameters	EBV+ *N* = 58 *N* (%)		EBV− *N* = 52 *N* (%)	Control Group *N* = 40 *N* (%)	*p* Value
EBVCA IgA IgG *p*	19 (32.7) 51 (87.9)	0.0007 *	- 32 (61.5)	- 26 (65.0)	0.0005 *
EBNA IgA IgG *p*	21 (36.2) 46 (79.3)	0.0017 *	- 25 (48.1)	- 19 (47.5)	0.0025 *
EA IgA IgG	5 (8.6) 17 (29.3)		- -	- -	
EBVCA IgM	-		-	-	

* statistically significant Pearson’s chi-square test; Fischer’s exact test.

**Table 3:** The level of selected anti-EBV antibodies in the IgA and IgG classes in EBV positive and EBV negative OPSCC patients, as well as in the control group (U/mL).

Antibodies U/mL	Group	Mean	Median	Minimum	Maximum	SD	*p* Value
EBVCA IgG EBVCA IgA	EBV+ EBV− Control EBV+	775.2 514.6 512.0 505.8	837.5 515.4 515.4 456.9	248.9 385.2 385.2 243.6	980.9 622.3 622.4 923.6	188.7 60.37 66.75 197.9	<0.0001 *
EBNA1 IgG EBNA1 IgA	EBV+ EBV− Control EBV+ EBV− Control	487.0 375.6 357.0 683.2 - -	490.9 360.2 320.8 745.9 - -	230.4 290.5 280.5 260.5 - -	655.1 562.1 465.8 902.5 - -	117.3 68.56 68.87 230.7 - -	<0.0001 *
EA IgA EA IgG	EBV+ EBV− Control EBV+ EBV− Control	392.0 - - 450.0 - -	500.0 - - 395.0 - -	213.0 - - 234.0 - -	521.0 - - 948.0 - -	160.0 - - 195.1 - -	

* Statistically significant; Kruskal–Wallis Test.

**Table 4:** The prevalence of anti-EBNA IgA, EBNA IgG, EBVCA IgA, and EBVCA IgG antibodies in EBV positive OPSCC patients in relation to grade (G1–G3) (%).

Antibodies (%)	G1 *N* = 19 *N* (%)	G2 *N* = 30 *N* (%)	G3 *N* = 9 *N* (%)	*p* Value
EBNA IgA	4 (21.5)	9 (30.0)	8 (88.9)	0.002 *
EBNA IgG	8 (42.1)	29 (96.7)	9 (100.0)	<0.001 *
EBVCA IgA	3 (15.7)	13 (43.3)	8 (88.9)	0.125
EBVCA IgG	18 (94.7)	24 (80.0)	9 (100.0)	0.193

* statistically significant; Pearson’s chi-square test; Fischer’s exact test.

**Table 5:** The prevalence of anti-EBNA IgA, EBNA IgG, EBVCA IgA, and EBVCA IgG antibodies in OPSCC patients depending on the TNM classification (%).

Antibodies (%)	T1 *N* = 7 *N* (%)	T2 *N* = 27 *N* (%)	T3 *N* = 16 *N* (%)	T4 *N* = 8 *N* (%)	*p* Value
EBNA IgA	1 (14.3)	6 (22.2)	7 (43.7)	7 (87.5)	0.004 *
IgG	2 (28.6)	20 (74.1)	16 (100.0)	8 (100.0)	<0.001
EBVCA IgA	0	11 (40.7)	6 (37.5)	2 (25.0)	0.212
IgG	7 (100.0)	19 (74.0)	16 (100.0)	8 (100.0)	0.037 *
**Antibodies**	**N0** ***N* = 23** ***N* (%)**	**N1** ***N* = 11** ***N* (%)**	**N2** **N = 14** **N (%)**	**N3** ***N* = 10** ***N* (%)**	***p* Value**
EBNA IgA	6 (26.1)	1 (9.1)	5 (35.7)	9 (90.0)	0.001 *
IgG	12 (52.1)	10 (90.9)	14 (100.0)	10 (100.0)	<0.001 *
EBVCA IgA	4 (17.4)	7 (63.6)	5 (35.7)	3 (30.0)	0.071
IgG	20 (86.9)	7 (63.6)	14 (100.0)	10 (100.0)	0.029 *

* statistically significant; Pearson’s chi-square test; Fischer’s exact test.

**Table 6:** Prevalence of anti-Zta and anti-LMP1 antibodies in EBV positive and EBV negative oropharyngeal cancer patients.

Antibodies	EBV+ *N* = 58 *N* (%)	EBV− *N* = 52 *N* (%)	*p* Value
Zta IgA	33 (56.8)	9 (17.3)	<0.0001 *
Zta IgG	31 (53.4)	6 (11.5)	<0.0001 *
LMP1 IgA	21 (36.2)	-	
LMP1 IgG	32 (55.1)	-	

* statistically significant; Pearson’s chi-square test; Fischer’s exact test.

**Table 7:** The frequencies of anti-Zta and LMP1 antibodies in IgA and IgG classes in relation to grading (G1–G3).

Antibodies (%)	G1 *N* = 19 *N* (%)	G2 *N* = 30 *N* (%)	G3 *N* = 9 *N* (%)	*p* Value
Zta IgA	13 (68.4)	11 (36.7)	9 (100.0)	0.0011 *
IgG	10 (52.6)	12 (40.0)	8 (88.9)	0.0229 *
LMP1 IgA	8 (42.1)	9 (30.0)	4 (44.4)	0.6104
IgG	7 (36.8)	17 (56.7)	8 (88.9)	0.0346 *

* statistically significant; Pearson’s chi-square test; Fischer’s exact test.

**Table 8:** The frequencies of anti-Zta and LMP1 antibodies in IgA and IgG classes in relation to TNM classification.

Antibodies (%)	T1 *N* = 7 *N* (%)	T2 *N* = 27 *N* (%)	T3 *N* = 16 *N* (%)	T4 *N* = 8 *N* (%)	*p* Value
Zta IgA	5 (71.4)	13 (48.1)	4 (25.0)	7 (87.5)	0.0208 *
Zta IgG	2 (28.5)	13 (48.1)	8 (50.0)	8 (100.0)	0.0850
LMP1 IgA	3 (42.8)	11 (40.7)	4 (25.0)	5 (62.5)	0.3746
LMP1 IgG	2 (28.5)	12 (44.4)	2 (12.5)	5 (62.5)	0.1725
**Antibodies** **(%)**	**N0** ***N* = 23** ***N* (%)**	**N1** ***N* = 11** ***N* (%)**	**N2** ***N* = 14** ***N* (%)**	**N3** ***N* = 10** ***N* (%)**	***p* Value**
Zta IgA	8 (34.7)	0	4 (28.6)	10 (100.0)	<0.001 *
IgG	11 (47.8)	4 (36.4)	6 (42.8)	9 (90.0)	0.0532
LMP1 IgA	10 (43.4)	4 (36.4)	2 (14.3)	5 (50.0)	0.2293
IgG	7 (30.4)	7 (63.6)	9 (64.3)	9 (90.0)	0.0098 *

* statistically significant; Pearson’s chi-square test; Fischer’s exact test.

## Data Availability

Due to privacy and ethical concerns, the data used in this study are available from the corresponding author upon reasonable request.
